# Population based allele frequencies of disease associated polymorphisms in the Personalized Medicine Research Project

**DOI:** 10.1186/1471-2156-11-51

**Published:** 2010-06-17

**Authors:** Deanna S Cross, Lynn C Ivacic, Elisha L Stefanski, Catherine A McCarty

**Affiliations:** 1 Center for Human Genetics, Marshfield Clinic Research Foundation, 1000 N. Oak Ave., Marshfield, WI, USA

## Abstract

**Background:**

There is a lack of knowledge regarding the frequency of disease associated polymorphisms in populations and population attributable risk for many populations remains unknown. Factors that could affect the association of the allele with disease, either positively or negatively, such as race, ethnicity, and gender, may not be possible to determine without population based allele frequencies.

Here we used a panel of 51 polymorphisms previously associated with at least one disease and determined the allele frequencies within the entire Personalized Medicine Research Project population based cohort. We compared these allele frequencies to those in dbSNP and other data sources stratified by race. Differences in allele frequencies between self reported race, region of origin, and sex were determined.

**Results:**

There were 19544 individuals who self reported a single racial category, 19027 or (97.4%) self reported white Caucasian, and 11205 (57.3%) individuals were female. Of the 11,208 (57%) individuals with an identifiable region of origin 8337 or (74.4%) were German.

41 polymorphisms were significantly different between self reported race at the 0.05 level. Stratification of our Caucasian population by self reported region of origin revealed 19 polymorphisms that were significantly different (p = 0.05) between individuals of different origins. Further stratification of the population by gender revealed few significant differences in allele frequencies between the genders.

**Conclusions:**

This represents one of the largest population based allele frequency studies to date. Stratification by self reported race and region of origin revealed wide differences in allele frequencies not only by race but also by region of origin within a single racial group. We report allele frequencies for our Asian/Hmong and American Indian populations; these two minority groups are not typically selected for population allele frequency detection. Population wide allele frequencies are important for the design and implementation of studies and for determining the relevance of a disease associated polymorphism for a given population.

## Background

One of the challenges for translating disease associated polymorphisms into use is the lack of knowledge regarding the frequency of the polymorphism in the targeted population. Without this information, population attributable risk remains unknown. In addition, factors that could affect the association of the allele with disease, either positively or negatively, such as ethnicity and gender, may not be possible to determine without population based allele frequencies. There are a number of reasons that these frequencies have yet to be determined. Disease associations whether completed through candidate gene studies or genome wide studies are determined using case control studies that classify affected and unaffected individuals but are not necessarily representative of a population and may need correction for population stratification [1-5]. Another reason that many polymorphisms lack population allele frequencies is cost; without a clear necessity to genotype a large diverse population funding for such studies is scarce [6,7]. Finally, with the creation of large population wide biorepositories still in its infancy there has been a lack of samples for genotyping [8-11].

In the past, geneticists have relied on a small population of well characterized reference samples to estimate allele frequency in a population [12-15]. These samples include the HapMap collection of individuals with different ethnicities that has been anonomized and immortalized by Coriell [13,14]. These populations form the population allele frequencies most often used in dbSNP [15] and a subset of these samples are also used for population allele frequencies on the Cancer500 website [12]. While these population samples are a valuable resource, they were not collected to be representative of a population and are often made up of related individuals. Even with the advent of the 1000 genomes project [16], there will still be a relatively few samples of any ethnic background to determine population allele frequencies with any certainty. This lack of a representative population distribution may not lead to representative population allele frequencies [2,3,5,8,1]. Ethnicities, such as Hispanic, are not well represented within the dbSNP database with often less than 100 individuals genotyped to determine population allele frequencies. Other backgrounds such as Native American may be missing from dbSNP all together [15].

One of the most widely used population samples the Centre d'Etude du Polymorphisme Humain (CEPH) Human Diversity Panel, has been successful for determining large trends in human diversity and population structure [17-21], however even with the Caucasian population available from this Centre d'Etude du Polymorphisme Humain (CEPH) collection and other collections, there are too few individuals to determine allelic variation within an ethnic population. Recent studies have shown that even within the Central European population the country of origin can have a great impact on allele frequencies with clearly seen variation between countries and even within a country [22-24][Feder, 2008 #87;Hannelius, 2008 #107]. This creates an even more challenging problem when attempting to use these generalized allele frequencies in a given population to estimate the burden disease associated polymorphisms have on disease within a population [20,21]. Current genome wide association studies (GWAS) control for this by stratifying the individuals by ethnicity and country of origin but because of their case-control nature these studies are less informative regarding allele frequencies for an entire population group [2,3,5,8,1].

One method of determining population wide allele frequencies is to genotype entire biorepositories regardless of case or control status. There are several repositories that are population based, including NHANES III [25], the UK biobank [11], the Marshfield Clinic Research Foundation Personalized Medicine Research Project [26], the Kaiser Permanente Research Program on Genes Environment and Health [8], and the Vanderbilt Databank Resource [10]. These biorepositories offer a broad cross sectional population available for genotyping and will be a valuable resource as more genotypes become publically available from all of these resources.

Here we used a panel of polymorphisms previously associated with disease and used as quality control markers in the PMRP population [27] to determine the allele frequencies within the entire Personalized Medicine Research Project population based cohort (Additional File 1). These allele frequencies found in our population were then compared to the frequencies found in dbSNP and other data bases stratified by race, with some of these allele frequencies varying over 10% from the reported allele frequencies. Differences in allele frequencies between self reported ethnicities within our population and differences between sexes were determined as well.

## Results

### Population Characteristics

Demographic characteristics for the population are presented in Table 1. There were 19544 individuals who self reported a single racial category, of these individuals the vast majority (19027 or 97.4%) self reported white Caucasian. Of individuals who selected ancestral origin, 11,208 (57%) selected either a single ancestral origin or two ancestral origins that were grouped together for our analysis (ex. Norwegian and Swedish). Of the individuals in the analysis the majority self reported German origin (8337 or 74.4%). Females outnumbered males in our sample with 11205 (57.3%) individuals with confirmed female sex.

**Table 1:** Population demographics for individuals genotyped within the PMRP cohort

Self Reported Race/Ethnicity/Region of Ancestry	Number of participants	Sex	
		Male	Female
Self Reported	19544	8338	11206 (57.3%)
Race/Ethnicity			

White/Caucasian	19027 (97.35%)	8095	10932
White/Hispanic	206 (1.05%)	98	108
Black/African American	50 (0.26%)	31	19
Asian/Hmong	94 (0.48%)	36	58
American Indian	167 (0.85%)	69	98

			

Self Reported Region of Ancestry	11208		
Germany	8337 (74.38%)	3741	4596
Eastern Europe (Poland/Czech Republic)	780 (6.96%)	357	423
Scandinavia (Sweden/Norway)	653 (5.83%)	306	347
British and Irish Isles	1438 (12.83%)	638	800

### Population allele frequencies

**Figure 1:**
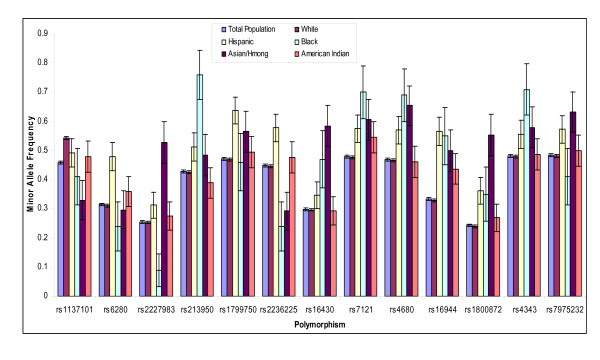
**Polymorphisms with major and minor alleles that vary with race**. Minor allele frequency for the total population and the same allele for each racial group, with 95% confidence intervals.

As expected our population allele frequencies were significantly different for different self reported races. Allele frequencies stratified by race are reported in Table 2. Of the 51 polymorphisms tested 41 were significantly different between self reported race at the 0.05 level with 35 polymorphisms significantly different with p values of < 0.0001 (Table 2). Of these polymorphisms, 13 exhibited a minor allele frequency of over 50% within a racial category although the limited size of the non-Caucasian groups in this study creates a large amount of uncertainty regarding the actual allele frequencies in these racially stratified populations (Figure 1). For alleles with greater than a 2% minor allele frequency, only two polymorphisms deviated from Hardy Weinberg equilibrium (p = 0.01), rs4680 in the COMT gene in Black/African American individuals and rs1800588 in the LIPC gene in individuals self reported as Hispanic.

**Table 2:** Population allele frequencies stratified by self reported race

Gene	Polymorphism	Allele (MA)	Population MAF (95% CI)	White/ Caucasian MAF (95% CI)	White Hispanic MAF (95% CI)	Black African American MAF (95% CI)	Asian Hmong MAF (95% CI)	American Indian MAF (95% CI)	P value
LEPR	rs1137101	G(A)	0.459 (0.454-0.464)	0.544 (0.539-0.549)	0.493 (0.445-0.541)	0.410 (0.314-0.506)	0.330 (0.263-0.397)	0.479 (0.425-0.533)	<0.0001
RNASEL	rs486907	G(A)	0.375 (0.370-0.380)	0.379 (0.374-0.384)	0.214 (0.174-0.254)	0.100 (0.041-0.159)	0.308 (0.242-0.374)	0.284 (0.236-0.332)	<0.0001
APOB	rs1042031	G(A)	0.180 (0.176-0.184)	0.18 (0.176-0.184)	0.167 (0.131-0.203)	0.220 (0.139-0.301)	0.063 (0.028-0.098)	0.182 (0.141-0.223)	0.0023
CTLA4	rs231775	A(G)	0.388 (0.383-0.393)	0.386 (0.381-0.391)	0.480 (0.432-0.528)	0.370 (0.275-0.465)	0.479 (0.408-0.550)	0.413 (0.360-0.466)	<0.0001
AGTR1	rs5186	A(C)	0.298 (0.293-0.303)	0.299 (0.294-0.304)	0.306 (0.262-0.350)	0.110 (0.049-0.171)	0.106 (0.062-0.150)	0.287 (0.238-0.336)	<0.0001
DRD3	rs6280	T(C)	0.315 (0.310-0.320)	0.311 (0.306-0.316)	0.480 (0.432-0.528)	0.240 (0.156-0.324)	0.297 (0.232-0.362)	0.359 (0.308-0.410)	<0.0001
FABP2	rs1799883	G(A)	0.262 (0.258-0.266)	0.261 (0.257-0.265)	0.293 (0.249-0.337)	0.240 (0.156-0.324)	0.250 (0.188-0.312)	0.295 (0.246-0.344)	0.3968
ADD1	rs4961	G(T)	0.192 (0.188-0.196)	0.191 (0.187-0.195)	0.209 (0.170-0.248)	0.100 (0.041-0.159)	0.346 (0.278-0.414)	0.165 (0.125-0.205)	<0.0001
ADRB2	rs1042714	C(G)	0.428 (0.423-0.433)	0.433 (0.428-0.438)	0.214 (0.174-0.254)	0.250 (0.165-0.335)	0.170 (0.116-0.224)	0.404 (0.351-0.457)	<0.0001
FGFR4	rs351855	C(T)	0.305 (0.300-0.310)	0.303 (0.298-0.308)	0.430 (0.382-0.478)	0.170 (0.096-0.244)	0.436 (0.365-0.507)	0.314 (0.264-0.364)	<0.0001
EDN1	rs5370	G(T)	0.213 (0.209-0.217)	0.214 (0.210-0.218)	0.182 (0.145-0.219)	0.180 (0.105-0.255)	0.255 (0.193-0.317)	0.165 (0.125-0.205)	0.0835
HTR1B	rs6296	G(C)	0.270 (0.266-0.274)	0.268 (0.264 -0.272)	0.391 (0.344-0.438)	0.220 (0.139-0.301)	0.367 (0.298-0.436)	0.320 (0.270-0.370)	<0.0001
EGFR	rs2227983	G(A)	0.255 (0.251-0.259)	0.253 (0.249-0.257)	0.313 (0.268-0.358)	0.090 (0.034-0.146)	0.527 (0.456-0.598)	0.275 (0.227-0.323)	<0.0001
CFTR	rs213950	G(A)	0.428 (0.423-0.433)	0.426 (0.421-0.431)	0.512 (0.464-0.560)	0.760 (0.676-0.844)	0.484 (0.413-0.555)	0.389 (0.337-0.441)	<0.0001
PON2	rs7493	C(G)	0.244 (0.240-0.248)	0.244 (0.240-0.248)	0.231 (0.190-0.272)	0.350 (0.257-0.443)	0.245 (0.184-0.306)	0.207 (0.164-0.250)	0.2519
LPL	rs328	C(G)	0.098 (0.095-0.101)	0.098 (0.095-0.101)	0.083 (0.056-0.110)	0.080 (0.027-0.133)	0.080 (0.041-0.119)	0.108 (0.075-0.141)	0.9200
9p21	rs2383206	A(G)	0.500 (0.495-0.505)	0.499 (0.494-0.504)	0.551 (0.503-0.599)	0.550 (0.452-0.648)	0.505 (0.434-0.576)	0.547 (0.494-0.600)	0.2803
RET	rs1800861	A(C)	0.233 (0.229-0.237)	0.232 (0.228-0.236)	0.218 (0.178-0.258)	0.130 (0.064-0.196)	0.452 (0.381-0.523)	0.219 (0.175-0.263)	<0.0001
ADRB1	rs1801253	C(G)	0.270 (0.266-0.274)	0.271 (0.267-0.275)	0.163 (0.127-0.199)	0.500 (0.402-0.598)	0.229 (0.169-0.289)	0.222 (0.177-0.267)	<0.0001
PLAU	rs2227564	C(T)	0.247 (0.243-0.251)	0.247 (0.243-0.251)	0.250 (0.208-0.292)	0.100 (0.041-0.159)	0.299 (0.234-0.364)	0.246 (0.200-0.292)	0.354
MMP1	rs1799750	-(G)	0.471 (0.466-0.476)	0.469 (0.464-0.474)	0.638 (0.592-0.684)	0.460 (0.362-0.558)	0.565 (0.494-0.636)	0.494 (0.440-0.548)	<0.0001
VWF	rs1063856	A(G)	0.359 (0.354-0.364)	0.361 (0.356-0.366)	0.250 (0.208-0.292)	0.490 (0.392-0.588)	0.170 (0.116-0.224)	0.290 (0.241-0.339)	<0.0001
HTR2A	rs6313	C(T)	0.406 (0.401-0.411)	0.406 (0.401-0.411)	0.369 (0.322-0.416)	0.350 (0.257-0.443)	0.450 (0.379-0.521)	0.392 (0.340-0.444)	0.3286
MTHFD1	rs2236225	C(T)	0.448 (0.443-0.453)	0.447 (0.442-0.452)	0.578 (0.530-0.626)	0.240 (0.156-0.324)	0.293 (0.228-0.358)	0.476 (0.422-0.530)	<0.0001
LIPC	rs1800588	C(T)	0.218 (0.214-0.222)	0.214 (0.210-0.218)	0.480 (0.432-0.528)	0.430 (0.333-0.527)	0.346 (0.278-0.414)	0.254 (0.207-0.301)	<0.0001
MMP2	rs243865	C(T)	0.246 (0.242-0.250)	0.247 (0.243-0.251)	0.218 (0.178-0.258)	0.100 (0.041-0.159)	0.112 (0.067-0.157)	0.257 (0.210-0.304)	<0.0001
CYBA	rs4673	C(T)	0.339 (0.334-0.344)	0.341 (0.336-0.346)	0.209 (0.170-0.248)	0.370 (0.275-0.465)	0.213 (0.154-0.272)	0.371 (0.319-0.423)	<0.0001
CETP	rs708272	C(T)	0.421 (0.416-0.426)	0.422 (0.417-0.427)	0.432 (0.384-0.480)	0.306 (0.216-0.396)	0.398 (0.328-0.468)	0.428 (0.375-0.481)	0.0924
ELAC2	rs4792311	G(A)	0.303 (0.298-0.308)	0.304 (0.299-0.309)	0.262 (0.220-0.304)	0.280 (0.192-0.368)	0.165 (0.112-0.218)	0.317 (0.267-0.367)	<0.0001
ENOSF1/TYMS	rs16430	+(-)	0.299 (0.294-0.304)	0.297 (0.292-0.302)	0.347 (0.301-0.393)	0.470 (0.372-0.568)	0.585 (0.515-0.655)	0.293 (0.244-0.342)	<0.0001
FUT2	rs601338	G(A)		0.456 (0.451-0.461)	0.267 (0.224-0.310)	0.500 (0.402-0.598)	0.133 (0.084-0.182)	0.410 (0.357-0.463)	<0.0001
LDLR	rs688	C(T)	0.422 (0.417-0.427)	0.423 (0.418-0.428)	0.425 (0.377-0.473)	0.150 (0.080-0.220)	0.335 (0.268-0.402)	0.467 (0.413-0.521)	<0.0001
GNAS	rs7121	C(T)	0.480 (0.475-0.485)	0.477 (0.472-0.482)	0.575 (0.527-0.623)	0.700 (0.610-0.790)	0.606 (0.536-0.676)	0.545 (0.492-0.598)	<0.0001
CBS	rs234706	G(A)	0.345 (0.340-0.350)	0.348 (0.343-0.353)	0.233 (0.192-0.274)	0.220 (0.139-0.301)	0.118 (0.072-0.164)	0.335 (0.284-0.386)	<0.0001
IL1B	rs16944	G(A)	0.334 (0.329-0.339)	0.329 (0.324-0.334)	0.566 (0.518-0.614)	0.550 (0.452-0.648)	0.500 (0.429-0.571)	0.437 (0.384-0.490)	<0.0001
NOS3	rs1799983	G(T)	0.306 (0.301-0.311)	0.308 (0.303-0.313)	0.228 (0.187-0.269)	0.130 (0.064-0.196)	0.149 (0.098-0.200)	0.263 (0.216-0.310)	<0.0001
TGFB1	rs1800469	C(T)	0.308 (0.303-0.313)	0.306 (0.301-0.311)	0.468 (0.420-0.516)	0.250 (0.165-0.335)	0.410 (0.340-0.480)	0.287 (0.238-0.336)	<0.0001
TNFa	rs1800629	G(A)	0.170 (0.166-0.174)	0.171 (0.167-0.175)	0.083 (0.056-0.110)	0.160 (0.088-0.232)	0.080 (0.041-0.119)	0.132 (0.096-0.168)	<0.0001
IL6	rs1800795	G(C)	0.434 (0.429-0.439)	0.440 (0.435-0.445)	0.199 (0.160-0.238)	0.160 (0.088-0.232)	0.085 (0.045-0.125)	0.326 (0.276-0.376)	<0.0001
IL6	rs1800796	G(C)	0.057 (0.055-0.059)	0.052 (0.050-0.054)	0.260 (0.218-0.302)	0.110 (0.049-0.171)	0.479 (0.408-0.550)	0.111 (0.077-0.145)	<0.0001
IL10	rs1800872	C(A)	0.244 (0.240-0.248)	0.240 (0.236-0.244)	0.362 (0.316-0.408)	0.350 (0.257-0.443)	0.553 (0.482-0.624)	0.269 (0.221-0.317)	<0.0001
MTHFR	rs1801133	C(T)	0.327 (0.322-0.332)	0.327 (0.322-0.332)	0.485 (0.437-0.533)	0.100 (0.041-0.159)	0.229 (0.169-0.289)	0.344 (0.293-0.395)	<0.0001
LPL	rs268	A(G)	0.020 (0.019-0.021)	0.020 (0.019-0.021)	0.001 (0.000-0.004)	0	0	0.015 (0.002-0.028)	NA
ACE	rs4291	A(T)	0.377 (0.372-0.382)	0.389 (0.384-0.394)	0.415 (0.367-0.463)	0.347 (0.254-0.440)	0.316 (0.250-0.382)	0.377 (0.325-0.429)	0.2086
ACE	rs4343	G(A)	0.481 (0.476-0.486)	0.479 (0.474-0.484)	0.556 (0.508-0.604)	0.710 (0.621-0.799)	0.580 (0.509-0.651)	0.488 (0.434-0.542)	<0.0001
APOE	rs429358	T(C)	0.144 (0.141-0.147)	0.145 (0.141-0.149)	0.100 (0.071-0.129)	0.220 (0.139-0.301)	0.085 (0.045-0.125)	0.123 (0.088-0.158)	0.0108
APOE	rs7412	C(T)	0.083 (0.080-0.086)	0.083 (0.080-0.086)	0.030 (0.014-0.046)	0.140 (0.072-0.208)	0.060 (0.026-0.094)	0.084 (0.054-0.114)	0.0056
COMT	rs4680	A(G)	0.469 (0.464-0.474)	0.466 (0.461-0.471)	0.570 (0.522-0.618)	0.690 (0.599-0.781)	0.654 (0.586-0.722)	0.461 (0.408-0.514)	<0.0001
VDR	rs7975232	A(C)	0.485 (0.480-0.490)	0.483 (0.478-0.488)	0.573 (0.525-0.621)	0.410 (0.314-0.506)	0.633 (0.564-0.702)	0.500 (0.446-0.554)	<0.0001
VDR	rs731236	T(C)	0.384 (0.379-0.389)	0.387 (0.382-0.392)	0.245 (0.203-0.287)	0.320 (0.229-0.411)	0.165 (0.112-0.218)	0.359 (0.308-0.410)	<0.0001
VDR	rs1544410	G(A)	0.389 (0.384-0.394)	0.392 (0.387-0.397)	0.243 (0.202-0.284)	0.310 (0.219-0.401)	0.223 (0.163-0.283)	0.344 (0.293-0.395)	<0.0001

**Figure 2:**
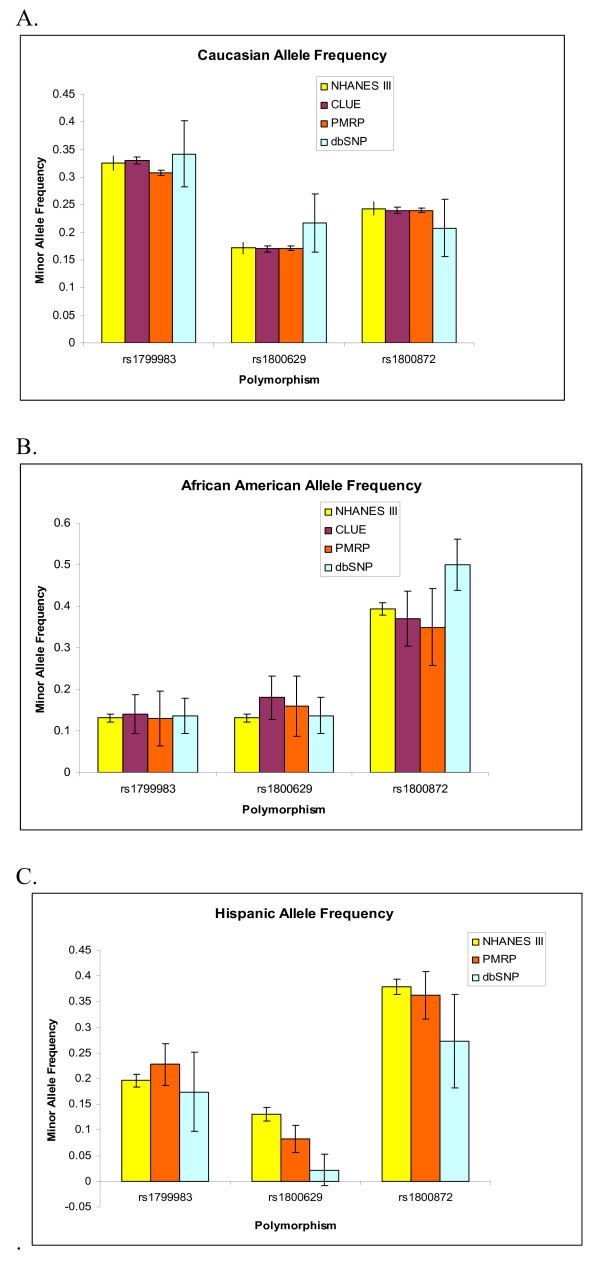
**Minor allele frequencies of 3 polymorphisms tested in different National populations stratified by race**. Comparison populations include NHANES III, CLUE II, PMRP, and dbSNP. A. Caucasian population B. African American Population C. Hispanic Population. Minor allele frequencies include 95% confidence intervals for the population minor allele frequency.

We compared the allele frequencies of our population with two previously published population allele frequencies, the CLUE II population [28] and the NHANES III population [29] as well as comparing our allele frequencies to those of dbSNP [15]and Cancer500 [28] allele frequencies. There were three polymorphisms assayed in all four populations, and when compared, our allele frequencies varied little from previously published population allele frequencies (Figure 2). Our Caucasian allele frequencies were within 2% of the NHANES III [29] and CLUE II [28] allele frequencies and our African American and Hispanic allele frequencies we were within 5% of these previously reported populations. In contrast, the allele frequencies reported here showed more variability when compared to reported allele frequencies in dbSNP [15]. When we compared all of the allele frequencies with those reported in the dbSNP [15] and Cancer500 [12] websites several polymorphisms differed by more than 10%. (Additional File 2). Unfortunately our American Indian population could not be compared as this is not one of the populations with widely reported allele frequencies.

### Allele frequencies by self reported region of ancestry

**Figure 3:**
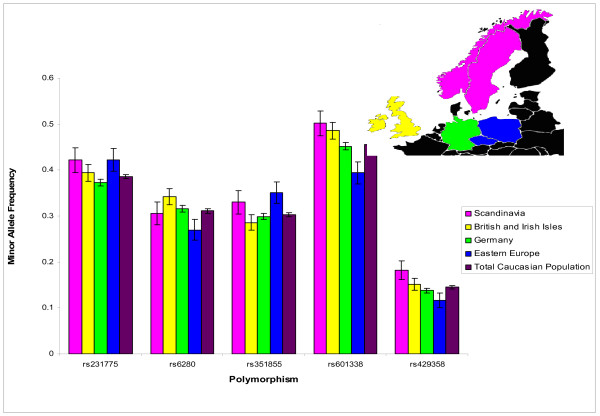
**Minor allele frequencies that vary significantly by region of origin**. Minor allele frequency for the Caucasian population and the same allele for each region of origin, with 95% confidence intervals.

There is mounting evidence that further population stratifications may be necessary as even within a racial group there can be significant differences among people of different ancestral origin. To investigate this, we stratified our Caucasian population by self reported region of origin to determine if there were differences in allele frequencies between individuals. 11,205 (57%) individuals could be categorized by a region of origin. Using this population, there were 19 polymorphisms that were significantly different (p = 0.05) between individuals of different origins (Table 3). Of these 5 (rs231775, rs6280, rs351855, rs601338, and rs429358) were significantly different with a p value of 0.0001 or less. Interestingly, the allele frequencies of some of the ethnic groups fell outside the 95% confidence interval of the total Caucasian MAF particularly the Eastern European ethnicity (Figure 3, Table 3).

**Table 3:** Population Caucasian allele frequencies stratified by self reported region of ancestry

Gene	Polymorphism	Allele (MA)	White Caucasian MAF (95% CI)	British and Irish Isles MAF (95% CI)	Eastern Europe MAF (95% CI)	Germany MAF (95% CI)	Scandinavia MAF (95% CI)	P value
LEPR	rs1137101	G(A)	0.544 (0.539-0.549)	0.554 (0.536-0.572)	0.523 (0.498-0.548)	0.544 (0.536-0.552)	0.500 (0.473-0.527)	0.032
RNASEL	rs486907	G(A)	0.379 (0.374-0.384)	0.363 (0.345-0.381)	0.381 (0.357-0.405)	0.384 (0.377-0.391)	0.376 (0.350-0.402)	0.553
APOB	rs1042031	G(A)	0.18 (0.176-0.184)	0.190 (0.176-0.204)	0.179 (0.160-0.198)	0.184 (0.178-0.190)	0.153 (0.133-0.173)	0.143
CTLA4	rs231775	A(G)	0.386 (0.381-0.391)	0.394 (0.376-0.412)	0.422 (0.397-0.447)	0.373 (0.366-0.380)	0.422 (0.395-0.449)	<0.0001
AGTR1	rs5186	A(C)	0.299 (0.294-0.304)	0.298 (0.281-0.315)	0.285 (0.263-0.307)	0.305 (0.298-0.312)	0.278 (0.254-0.302)	0.185
DRD3	rs6280	T(C)	0.311 (0.306-0.316)	0.342 (0.325-0.359)	0.270 (0.248-0.292)	0.316 (0.309-0.323)	0.306 (0.281-0.331)	0.0001
FABP2	rs1799883	G(A)	0.261 (0.257-0.265)	0.261 (0.245-0.277)	0.258 (0.236-0.280)	0.261 (0.254-0.268)	0.271 (0.247-0.295)	0.871
ADD1	rs4961	G(T)	0.191 (0.187-0.195)	0.188 (0.174-0.202)	0.165 (0.147-0.183)	0.192 (0.186-0.198)	0.197 (0.175-0.219)	0.158
ADRB2	rs1042714	C(G)	0.433 (0.428-0.438)	0.457 (0.439-0.475)	0.403 (0.379-0.427)	0.428 (0.420-0.436)	0.419 (0.392-0.446)	0.0018
FGFR4	rs351855	C(T)	0.303 (0.298-0.308)	0.286 (0.269-0.303)	0.351 (0.327-0.375)	0.299 (0.292-0.306)	0.330 (0.304-0.356)	<0.0001
EDN1	rs5370	G(T)	0.214 (0.210-0.218)	0.218 (0.203-0.233)	0.193 (0.173-0.213)	0.210 (0.204-0.216)	0.201 (0.179-0.223)	0.524
HTR1B	rs6296	G(C)	0.268 (0.264-0.272)	0.266 (0.250-0.282)	0.281 (0.259-0.303)	0.264 (0.257-0.271)	0.290 (0.265-0.315)	0.045
EGFR	rs2227983	G(A)	0.253 (0.249-0.257)	0.265 (0.249-0.281)	0.260 (0.238-0.282)	0.250 (0.243-0.257)	0.260 (0.236-0.284)	0.209
CFTR	rs213950	G(A)	0.426 (0.421-0.431)	0.423 (0.405-0.441)	0.444 (0.419-0.469)	0.427 (0.419-0.435)	0.424 (0.397-0.451)	0.085
PON2	rs7493	C(G)	0.244 (0.240-0.248)	0.252 (0.236-0.268)	0.279 (0.257-0.301)	0.239 (0.233-0.245)	0.257 (0.233-0.281)	0.004
LPL	rs328	C(G)	0.098 (0.095-0.101)	0.099 (0.088-0.110)	0.092 (0.078-0.106)	0.099 (0.094-0.104)	0.090 (0.074-0.106)	0.682
9p21	rs2383206	A(G)	0.499 (0.494-0.504)	0.522 (0.504-0.540)	0.496 (0.471-0.521)	0.495 (0.487-0.503)	0.477 (0.450-0.504)	0.150
RET	rs1800861	A(C)	0.232 (0.228-0.236)	0.213 (0.198-0.228)	0.256 (0.234-0.278)	0.240 (0.234-0.246)	0.203 (0.181-0.225)	0.001
ADRB1	rs1801253	C(G)	0.271 (0.267-0.275)	0.264 (0.248-0.280)	0.269 (0.247-0.291)	0.270 (0.263-0.277)	0.275 (0.251-0.299)	0.922
PLAU	rs2227564	C(T)	0.247 (0.243-0.251)	0.239 (0.223-0.255)	0.219 (0.198-0.240)	0.244 (0.237-0.251)	0.277 (0.253-0.301)	0.012
MMP1	rs1799750	-(G)	0.469 (0.464-0.474)	0.486 (0.468-0.504)	0.458 (0.433-0.483)	0.460 (0.452-0.468)	0.505 (0.478-0.532)	0.020
VWF	rs1063856	A(G)	0.361 (0.356-0.366)	0.381 (0.363-0.399)	0.337 (0.314-0.360)	0.353 (0.346-0.360)	0.372 (0.346-0.398)	0.006
HTR2A	rs6313	C(T)	0.406 (0.401-0.411)	0.400 (0.382-0.418)	0.370 (0.346-0.394)	0.412 (0.405-0.419)	0.397 (0.370-0.424)	0.071
MTHFD1	rs2236225	C(T)	0.447 (0.442-0.452)	0.456 (0.438-0.474)	0.442 (0.417-0.467)	0.446 (0.438-0.454)	0.439 (0.412-0.466)	0.792
LIPC	rs1800588	C(T)	0.214 (0.210-0.218)	0.225 (0.210-0.240)	0.232 (0.211-0.253)	0.213 (0.207-0.219)	0.212 (0.190-0.234)	0.077
MMP2	rs243865	C(T)	0.247 (0.243-0.251)	0.242 (0.226-0.258)	0.257 (0.235-0.279)	0.243 (0.236-0.250)	0.248 (0.225-0.271)	0.888
CYBA	rs4673	C(T)	0.341 (0.336-0.346)	0.318 (0.301-0.335)	0.365 (0.341-0.389)	0.338 (0.331-0.345)	0.325 (0.300-0.350)	0.083
CETP	rs708272	C(T)	0.422 (0.417-0.427)	0.436 (0.418-0.454)	0.409 (0.385-0.433)	0.420 (0.413-0.427)	0.436 (0.409-0.463)	0.027
ELAC2	rs4792311	G(A)	0.304 (0.299-0.309)	0.291 (0.274-0.308)	0.304 (0.281-0.327)	0.309 (0.302-0.316)	0.292 (0.267-0.317)	0.492
ENOSF1/TYMS	rs16430	+(-)	0.297 (0.292-0.302)	0.301 (0.284-0.318)	0.273 (0.251-0.295)	0.300 (0.293-0.307)	0.305 (0.280-0.330)	0.314
FUT2	rs601338	G(A)	0.456 (0.451-0.461)	0.486 (0.468-0.504)	0.394 (0.370-0.418)	0.452 (0.444-0.460)	0.502 (0.475-0.529)	<0.0001
LDLR	rs688	C(T)	0.423 (0.418-0.428)	0.443 (0.425-0.461)	0.406 (0.382-0.430)	0.421 (0.414-0.428)	0.420 (0.393-0.447)	0.038
GNAS	rs7121	C(T)	0.477 (0.472-0.482)	0.500 (0.482-0.518)	0.468 (0.443-0.493)	0.475 (0.467-0.483)	0.473 (0.446-0.500)	0.317
CBS	rs234706	G(A)	0.348 (0.343-0.353)	0.342 (0.325-0.359)	0.331 (0.308-0.354)	0.347 (0.340-0.354)	0.344 (0.318-0.370)	0.266
IL1B	rs16944	G(A)	0.329 (0.324-0.334)	0.343 (0.326-0.360)	0.326 (0.303-0.349)	0.332 (0.325-0.339)	0.312 (0.287-0.337)	0.170
NOS3	rs1799983	G(T)	0.308 (0.303-0.313)	0.318 (0.301-0.335)	0.287 (0.265-0.309)	0.309 (0.302-0.316)	0.296 (0.271-0.321)	0.420
TGFB1	rs1800469	C(T)	0.306 (0.301-0.311)	0.299 (0.282-0.316)	0.320 (0.297-0.343)	0.309 (0.302-0.316)	0.296 (0.271-0.321)	0.742
TNFa	rs1800629	G(A)	0.171 (0.167-0.175)	0.189 (0.175-0.203)	0.156 (0.138-0.174)	0.169 (0.163-0.175)	0.172 (0.152-0.192)	0.009
IL6	rs1800795	G(C)	0.440 (0.435-0.445)	0.430 (0.412-0.448)	0.453 (0.428-0.478)	0.438 (0.430-0.446)	0.455 (0.428-0.482)	0.200
IL6	rs1800796	G(C)	0.052 (0.050-0.054)	0.055 (0.047-0.063)	0.063 (0.051-0.075)	0.051 (0.048-0.054)	0.045 (0.034-0.056)	0.108
IL10	rs1800872	C(A)	0.240 (0.236-0.244)	0.236 (0.220-0.252)	0.242 (0.221-0.263)	0.240 (0.234-0.246)	0.244 (0.221-0.267)	0.551
MTHFR	rs1801133	C(T)	0.327 (0.322-0.332)	0.322 (0.305-0.339)	0.307 (0.284-0.330)	0.327 (0.320-0.334)	0.325 (0.300-0.350)	0.677
LPL	rs268	A(G)	0.020 (0.019-0.021)	0.020 (0.015-0.025)	0.017 (0.011-0.023)	0.021 (0.019-0.023)	0.032 (0.022-0.042)	0.034
ACE	rs4291	A(T)	0.377 (0.372-0.382)	0.377 (0.359-0.395)	0.373 (0.349-0.397)	0.379 (0.372-0.386)	0.379 (0.353-0.405)	0.879
ACE	rs4343	G(A)	0.479 (0.474-0.484)	0.480 (0.462-0.498)	0.506 (0.481-0.531)	0.476 (0.468-0.484)	0.488 (0.461-0.515)	0.009
APOE	rs429358	T(C)	0.145 (0.141-0.149)	0.151 (0.138-0.164)	0.117 (0.101-0.133)	0.138 (0.133-0.143)	0.182 (0.161-0.203)	<0.0001
APOE	rs7412	C(T)	0.083 (0.080-0.086)	0.077 (0.067-0.087)	0.082 (0.068-0.096)	0.089 (0.085-0.093)	0.076 (0.062-0.090)	0.299
COMT	rs4680	A(G)	0.466 (0.461-0.471)	0.470 (0.452-0.488)	0.484 (0.459-0.509)	0.467 (0.459-0.475)	0.421 (0.394-0.448)	0.009
VDR	rs7975232	A(C)	0.483 (0.478-0.488)	0.481 (0.463-0.499)	0.491 (0.466-0.516)	0.488 (0.480-0.496)	0.468 (0.441-0.495)	0.113
VDR	rs731236	T(C)	0.387 (0.382-0.392)	0.380 (0.362-0.398)	0.365 (0.341-0.389)	0.382 (0.375-0.389)	0.384 (0.358-0.410)	0.284
VDR	rs1544410	G(A)	0.392 (0.387-0.397)	0.387 (0.369-0.405)	0.368 (0.344-0.392)	0.387 (0.380-0.394)	0.387 (0.361-0.413)	0.479

### Allele frequencies by gender

Further stratification of the population by gender revealed few significant differences in allele frequencies between the genders. Only three polymorphisms were different in the total population p < 0.05 rs7121 (GNAS), rs1801253 (ADRB1) and rs1042714 (ADRB2). After stratification by racial group, these three polymorphisms were associated with gender differences in the white racial group. Three different polymorphisms exhibited gender differences in black individuals. Hispanic genders were different in two polymorphisms. Among the American Indian population 5 polymorphisms were different between genders and the Asian/Hmong population exhibited the greatest number of polymorphisms with gender differences with 7 polymorphisms. (Additional File 3).

## Discussion

This study determined the allele frequencies of 51 polymorphisms, previously associated with at least one disease state, in a large rural population. This represents one of the largest population based allele frequency studies to date. Stratification by self reported race and region of origin revealed wide differences in allele frequencies not only by race but also by region of origin within a single racial group. Here we report allele frequencies for our Asian/Hmong and American Indian populations. These two minority groups are not typically selected for population allele frequency detection. Stratification of our Caucasian population by region of origin revealed significantly different allele frequencies within a single racial group. As we move from gene disease discovery to the application of genetic knowledge, the true population wide allele frequencies become more important for the design and implementation of studies and for determining the relevance of a disease associated polymorphism for a given population.

Several recent studies have reported population wide allele frequencies; our study complements these previously published studies and contributes new information. The two largest US population allele frequency studies NHANES III [29]and CLUE II [28] also chose to report allele frequencies for polymorphisms associated with disease. The NHANES III study published allele frequencies from a nationally representative cohort for 91 polymorphisms previously associated with disease [29]; our study included 7 of these polymorphisms. The CLUE II population was genotyped for 49 polymorphisms in inflammatory genes [28], of which we also report allele frequencies for 4 of these polymorphisms. We chose to compare allele frequencies for the 3 polymorphisms all studies had in common. Our reported allele frequencies were very similar with 2 polymorphisms varying less than 1% between the studies, particularly when comparing the Caucasian population. This similarity in allele frequencies is contrasted with the dbSNP allele frequencies [15] which we varied from by over 10% for a number of polymorphisms. This highlights the continued need for large population based genotyping efforts to determine allele frequencies of disease associated polymorphisms.

Beyond agreeing with previously published population allele frequencies, this study reports allele frequencies for 43 polymorphisms not genotyped in either the NHANES III [29] or CLUE II populations [28]. The additional polymorphisms included alleles that have been associated with a number of different diseases such as cancer, heart disease, and diabetes [27] and will contribute to our understanding of disease risk. We also report initial population allele frequencies for two additional minority groups that have importance for our population; the Asian/Hmong and American Indian populations. However our minority allele frequencies have a high degree of uncertainty given the small number of individuals within the racial categories (50 African Americans) and several alleles have zero in at least one homozygote category. Racial category was self reported by an individual and admixtures were not determined, a single racial group was chosen by the individual.

As expected, our minority population allele frequencies were significantly different from the Caucasian allele frequencies for the majority (79%) of the tested polymorphisms, with 25% of the tested polymorphisms switching major and minor alleles between races. These differences will need to be considered when determining TAG and causal variants for disease. As an example, one of the IL6 polymorphisms tested here, rs1800796, has had both the C and G allele associated with osteoporosis depending on the race of the individual [28,29]. In Asians and Caucasians the minor allele is reversed creating complexity for determining the causal variant [30,31]. The IL1 B polymorphism (rs16944) exhibits different allele frequencies between races as well and the same allele has been shown in meta-analysis to be protective for gastric cancer in Asians and a risk factor for Caucasians [32-34]. Wide differences in allele frequencies may also contribute to differences in disease prevalence between racial groups. For instance the cystic fibrosis has large racial disparities [35] and as expected we found large differences in the CFTR gene (rs213950) among different racial groups in our population.

Here, we also began to investigate further substructure of our population within a single racial category. Because of the low number of minority racial groups in our population we chose to investigate potential substructure within only our Caucasian population. Using self reported region of ancestry, we found that 37% of the tested polymorphisms were significantly different between individuals reporting different regions of ancestry. Recent substructure analysis of Icelandic [23], Swedish[24,36], and Ashkenazi Jewish populations [22,37] determined that substructure was present even in these seemingly homogenous populations and that the substructure has implications for disease risk [2-5,22-24,36]. This study is one of the largest US studies to stratify a population by region of origin and demonstrates the large heterogeneity of the population even within a single racial group. These differences in allele frequencies may need to be considered when designing association studies in the future. One weakness of our analysis is the inability to categorize almost half (43%) of our Caucasian population into a single region of origin, because of our inability to determine the admixture given the self reported nature of the questionnaire and our limited genotyping. We are currently examining the more than 4000 individuals with whole genome scans completed to determine the ethnicities and regions of origin with greater accuracy.

Stratification of the population by sex revealed few differences in allele frequency within our population. This is similar to previously reported sex stratifications of large populations. The three genes that exhibited sex differences in our population (GNAS, ADRB1 and ADRB2) have also demonstrated gene-gender interactions in other studies. Interestingly, ADRB1 and ADRB2 alleles are associated with differential gender responses in blood pressure [38], and in rats these genes interact with sex hormones in a differential manner [39]. The GNAS gene was associated with different responses to hip arthroplasty [40]. While allele frequency differences between the sexes may not be frequent when they occur this may be an indicator of potential gene-gender interactions for future research.

## Conclusions

Using a panel of polymorphisms originally designed to uniquely identify individuals within our biorepository we were able to construct population allele frequencies for the PMRP population. In doing so, we have highlighted the need to consider population substructure beyond race using a large Caucasian population. Stratification of the population may lead to increased study power for future association studies. The genotyping data is available to investigators using the PMRP resource both for identification purposes and as a resource for investigating gene disease interactions.

## Methods

### Study population and questionnaire

The Personalized Medicine Research Project is a population based cohort of approximately 20,000 individuals ages 18 and up residing within one of 19 zip codes surrounding Marshfield Wisconsin, USA, who have agreed to provide DNA, serum, and plasma samples to be linked with a dynamic medical record for research [26]. Individuals are eligible to participate in the project if they have had care within the Marshfield Clinic Healthcare System within the 3 years prior to enrolment in PMRP. The majority of recruitment was accomplished in the first 18 months of enrolment beginning in September of 2002. At enrolment each individual was asked to complete a brief questionnaire which included questions regarding self reported racial affiliations and self reported ancestry using the US census questions, as well as occupational and environmental exposure questions. The enrolment questionnaire is available on the PMRP website [41] and the resulting summary statistics for our population has been published elsewhere [26].

The racial, ethnic and sex distribution of this cohort has been described previously. Briefly, the current cohort is over 98% Caucasian and 57.44% are females, with a mean age of 47.5 at enrolment [26]. For this study, 19544 individuals who self reported a single racial category were examined to determine allele frequencies. Individuals were allowed to select more than one ancestral origin. For this study individuals were grouped into four regions of ancestral origins: England or Ireland, Norway or Sweden, Czech Republic or Poland, and Germany. Individuals who selected two disparate regions were excluded from our ancestral analysis due to our inability to determine admixture percentages. However, individuals who selected multiple countries of origin within the same region were included in the analysis. (As an example, an individual selecting ancestral origin from both Norway and Sweden would be used in the analysis but an individual selecting Ireland and Germany would be excluded).

Informed consent was obtained upon enrolment in PMRP and this study was approved by the Marshfield Clinic Human Subjects Protection Institutional Review Board.

### Genotyping

The entire population was genotyped with 2 multiplex panels for a total of 52 alleles, including a sex marker, using the proprietary Sequenom^® ^platform (Additional File 1). An initial panel of 36 alleles was genotyped to serve as a quality control and assurance panel. Each allele in the panel was previously associated with at least one disease and was reported to have at least a 20% minor allele frequency in Caucasians[27]. In addition, a separate panel of 15 alleles each with at least one association with disease in Caucasian populations and with no restriction on allele frequency was also genotyped in the entire cohort. Only individuals who achieved an 80% or greater call rate were used in the study. In addition, each polymorphism was required to achieve at least an 80% call rate. Individual genotypes were decided via a mixture of automated calls from Typer 3.4 ^® ^[42] and manual calls; manual calls were checked by multiple individuals to assure agreement with the genotype assessment. Each multiplexed plate contained 4 water controls and 6 CEPH controls with 2 duplicate samples to ensure plate to plate accuracy. 8 individuals were duplicated blindly within the genotyping plates and all previous allele calls were compared to the study allele calls to ensure consistency within the cohort. In addition over 4,000 individuals in the cohort have been independently assayed using the Illumina 660 whole genome chip [43]. Discrepancies between genotyping were resolved with sequencing.

### Statistical Analysis

Hardy-Weinberg equilibrium was determined and polymorphisms were said to be out of equilibrium if the expected and observed allele frequency were outside of the 99% Confidence interval 1 degree of freedom using a χ^2 ^test. Allele frequencies were compared to reported allele frequencies both as a summary and for self reported racial group. Differences in allele frequencies between self reported racial, ethnic groups were determined using chi-squared analysis and the 95% binomial confidence limits were reported. Differences between the sexes were determined using chi-squared analysis. Differences were reported as significant if P < 0.05.

## Authors' contributions

DC conceived of the study, participated in the design and implementation of the genotyping assays, supervised the study, created the data analysis plan, and drafted the manuscript. Has read and approved the final manuscript. LI participated in the design and implementation of the genotyping assays, performed the genotyping assays, analyzed the data for Hardy-Weinberg deviations, and created allele frequency tables for race and ethnicity. Has read and approved the final manuscript. ES performed the genotyping assays, analyzed the data for Hardy-Weinberg deviations, and created allele frequency tables for race and ethnicity. Has read and approved the final manuscript. CM participated in study conception and design, was responsible for summary data for population characteristics, participated in manuscript preparation and editing. Has read and approved the final manuscript.

## Supplementary Material

Additional file 1 **Description of the 51 polymorphisms genotyped for this study**. Polymorphism list and description. Description of the 51 polymorphisms genotyped for this study. Word Table Click here for file

Additional file 2 **Allele frequencies with 10% or greater variation from allele frequencies reported in dbSNP stratified by race**. Allele frequency variation from dbSNP by race. Table of allele frequencies that varied by more than 10% from previously reported allele frequencies in dbSNP stratified by self reported race. Word Table Click here for file

Additional file 3 **Polymorphisms with significant differences between genders when stratified by race**. Allele frequency stratified by race and gender. Polymorphisms with significant differences between genders when stratified by race. Word Table Click here for file
