# Supplementary material for: Population based allele frequencies of disease associated polymorphisms in the Personalized Medicine Research Project
Source: BMC Genet. 2010 Jun 17;11:51. doi: 10.1186/1471-2156-11-51 (PMC2908055; doi:10.1186/1471-2156-11-51)
Supplement: Additional file 1 — Description of the 51 polymorphisms genotyped for this study. Polymorphism list and description. Description of the 51 polymorphisms genotyped for this study. Word Table [file 1471-2156-11-51-S1.DOC]

Additional file 1 – Description of the 51 polymorphisms genotyped

| **Polymorphism** | **Chromosome** | **Gene** | **Diseases Associated with Polymorphisms or Gene** |
| --- | --- | --- | --- |
| rs1137101 | 1p31 | LEPR  GeneID:3953 | Obesity, Insulin Resistance, Non-Hodgkin’s lymphoma |
| rs1801133 | 1p36 | MTHFR  GeneID: 4524 | Asthma, hypertension, cancer |
| rs486907 | 1q25 | RNASEL  GeneID:6041 | Prostate cancer |
| rs1800872 | 1q31 | IL10  GeneID:3586 | Rheumatoid arthritis, cancer |
| rs16944 | 2q14 | IL1B  GeneID:3553 | Alzheimer’s disease, cancer |
| rs1042031 | 2p24 | APOB  GeneID:338 | Cardiovascular disease, Dislipidemia |
| rs231775 | 2q33 | CTLA4  GeneID:1493 | Multiple Sclerosis, Autoimmune Disease |
| rs5186 | 3q21 | AGTR1  GeneID:185 | Metabolic syndrome, Aortic aneurism, Left -ventricular hypertrophy |
| rs6280 | 3q13.3 | DRD3  GeneID:1814 | Schizophrenia |
| rs1799883 | 4q28 | FABP2  GeneID:2169 | Metabolic syndrome, Type 2 diabetes |
| rs4961 | 4p16.3 | ADD1  GeneID:118 | Hypertension, Coronary artery disease |
| rs1042714 | 5q31 | ADRB2  GeneID:154 | Obesity, COPD |
| rs351855 | 5q35.1 | FGFR4  GeneID:2264 | Cancer |
| rs1800629 | 6p21 | TNFa  GeneID:7124 | Cardiovascular disease, Coronary artery disease, cancer |
| rs5370 | 6p24 | EDN1  GeneID:1906 | Asthma, sleep apnea |
| rs6296 | 6q13 | HTR1B  GeneID:3351 | substance abuse |
| rs2227983 | 7p12.3 | EGFR  GeneID:1956 | Cancer |
| rs1800795 | 7p21 | IL6  GeneID:3569 | Infection, cancer, cardiovascular disease |
| rs1800796 | 7p21 | IL6  GeneID:3569 | Infection, cancer, cardiovascular disease |
| rs7493 | 7q21.3 | PON2  GeneID:5445 | Myocardial infarction |
| rs213950 | 7q31.2 | CFTR  GeneID:1080 | Cystic fibrosis |
| rs1799983 | 7q36 | NOS3  GeneID:4846 | Coronary artery disease, cancer |
| rs328 | 8p22 | LPL  GeneID: 4023 | Left ventricular hypertrophy |
| rs268 | 8p22 | LPL  GeneID: 4023 | Coronary artery disease |
| rs2383206 | 9p21 |  | Coronary artery disease |
| rs1800861 | 10q11.2 | RET  GeneID:5979 | Hirschsprung disease, Thyroid cancer |
| rs1801253 | 10q24 | ADRB1  GeneID:153 | Insulin Resistance |
| rs2227564 | 10q24 | PLAU  GeneID:5328 | Alzheimer’s disease, asthma |
| rs1799750 | 11q22.3 | MMP1  GeneID:4312 | Endometriosis, Osteolysis, Rheumatoid authritis |
| rs1063856 | 12p13.3 | VWF  GeneID:7450 | Hypertension |
| rs731236 | 12q13 | VDR  GeneID: 7421 | Diabetes, cardiovascular disease, osteoporosis, cancer |
| rs1544410 | 12q13 | VDR  GeneID: 7421 | Diabetes, cardiovascular disease, osteoporosis, cancer |
| rs7975232 | 12q13 | VDR  GeneID: 7421 | Diabetes, cardiovascular disease, osteoporosis, cancer |
| rs6313 | 13q14 | HTR2A  GeneID:3356 | Psychiatric disorders |
| rs2236225 | 14q24 | MTHFD1  GeneID:4522 | Neural tube defects |
| rs1800588 | 15q21 | LIPC  GeneID:3990 | Coronary artery disease |
| rs243865 | 16q13 | MMP2  GeneID:4313 | Cancer |
| rs4673 | 16q24 | CYBA  GeneID:1535 | Coronary artery disease |
| rs708272 | 16q21 | CETP  GeneID:1071 | Coronary artery disease |
| rs4291 | 17q23 | ACE  GeneID:1636 | Depression, Alzheimer’s disease |
| rs4343 | 17q23 | ACE  GeneID:1636 | Depression, Alzheimer’s disease, cardiovascular disease |
| rs4792311 | 17p11 | ELAC2  GeneID:60528 | Prostate cancer |
| rs16430 | 18p11.3 | ENOSF1/TYMS  GeneID:55556 | Cancer |
| rs429358 | 19q13 | APOE  GeneID: 348 | Alzheimer’s disease |
| rs7412 | 19q13 | APOE  GeneID: 348 | Alzheimer’s disease |
| rs1800469 | 19q13 | TGFB1  GeneID:7040 | Cardiovascular disease, cancer |
| rs601338 | 19q13.3 | FUT2  GeneID:2524 | Infection susceptibility |
| rs688 | 19p13.2 | LDLR  GeneID:3949 | Alzheimer’s disease, Coronary artery disease |
| rs7121 | 20q13.3 | GNAS  GeneID: 2778 | Obesity, Cancer |
| rs234706 | 21q22 | CBS  GeneID:875 | Oral cleft defects |
| rs4680 | 22q11.21 | COMT  GeneID:1312 | Schizophrenia, ADHD |

Table partially recreated from : [27] Cross DS, Ivacic LC, McCarty CA: **Development of a fingerprinting panel using medically relevant polymorphisms**. *BMC Med Genomics* 2009, **2**:17.
