# Supplementary material for: Population based allele frequencies of disease associated polymorphisms in the Personalized Medicine Research Project
Source: BMC Genet. 2010 Jun 17;11:51. doi: 10.1186/1471-2156-11-51 (PMC2908055; doi:10.1186/1471-2156-11-51)
Supplement: Additional file 2 — Allele frequencies with 10% or greater variation from allele frequencies reported in dbSNP stratified by race. Allele frequency variation from dbSNP by race. Table of allele frequencies that varied by more than 10% from previously reported allele frequencies in dbSNP stratified by self reported race. Word Table [file 1471-2156-11-51-S2.DOC]

**Alleles with an allele frequency at least 10% different than the allele frequency reported in dbSNP, stratified by race.**

Allele frequencies reported here are for the same allele reported in Table 2.

Caucasian Population

| Polymorphism | Allele Frequency (dbSNP) | Observed Minor Allele Frequency  (PMRP cohort) | % Difference |
| --- | --- | --- | --- |
| rs1799883 | 0.37 | 0.26 | 11% |
| rs1800588 | 0.33 | 0.21 | 12% |

**African/American Population**

| Polymorphism | Allele Frequency (dbSNP) | Observed Minor Allele Frequency  (PMRP cohort) | % Difference |
| --- | --- | --- | --- |
| rs213950 | 0.86 | 0.76 | 10% |
| rs1801253 | 0.29 | 0.50 | 21% |
| rs1800588 | 0.54 | 0.43 | 11% |
| rs16430 | 0.59 | 0.47 | 12% |
| rs7121 | 0.80 | 0.70 | 10% |
| rs1800795 | 0.02 | 0.16 | 14% |
| rs1800872 | 0.50 | 0.35 | 15% |
| rs429358 | 0.02 | 0.22 | 20% |
| rs1800469 | 0.15 | 0.25 | 10% |
| rs7975232 | 0.26 | 0.41 | 15% |

Hispanic

| Polymorphism | Allele Frequency (dbSNP) | Observed Minor Allele Frequency  (PMRP cohort) | % Difference |
| --- | --- | --- | --- |
| rs231775 | 0.20 | 0.48 | 28% |
| rs5370 | 0.02 | 0.18 | 16% |
| rs351855 | 0.58 | 0.43 | 15% |
| rs1800588 | 0.37 | 0.48 | 11% |
| rs243865 | 0.09 | 0.22 | 13% |
| rs4680 | 0.46 | 0.57 | 11% |
| rs1801133 | 0.31 | 0.49 | 28% |
| rs731236 | 0.35 | 0.25 | 10% |
| rs1544410 | 0.37 | 0.24 | 13% |

| Polymorphism | Allele Frequency (dbSNP) | Observed Minor Allele Frequency  (PMRP cohort) | % Difference |
| --- | --- | --- | --- |
| rs1137101 | 0.14 | 0.33 | 19% |
| rs6280 | 0.47 | 0.30 | 17% |
| rs4961 | 0.48 | 0.35 | 13% |
| rs1042714 | 0.06 | 0.17 | 11% |
| rs6313 | 0.56 | 0.45 | 11% |
| rs4792311 | 0.03 | 0.17 | 14% |
| rs601338 | 0.02 | 0.13 | 11% |
| rs688 | 0.15 | 0.34 | 19% |
| rs4680 | 0.31 | 0.65 | 34% |
| rs1800469 | 0.54 | 0.41 | 13% |

**Asian/Hmong**
