# Supplementary material for: Population based allele frequencies of disease associated polymorphisms in the Personalized Medicine Research Project
Source: BMC Genet. 2010 Jun 17;11:51. doi: 10.1186/1471-2156-11-51 (PMC2908055; doi:10.1186/1471-2156-11-51)
Supplement: Additional file 3 — Polymorphisms with significant differences between genders when stratified by race. Allele frequency stratified by race and gender. Polymorphisms with significant differences between genders when stratified by race. Word Table [file 1471-2156-11-51-S3.DOC]

**Polymorphisms with significant differences between genders when stratified by race.**

Caucasian Population

| Polymorphism | Male Minor Allele Frequency | Female Minor Allele Frequency | p-value |
| --- | --- | --- | --- |
| rs7121 | 0.480 | 0.475 | 0.037 |
| rs1801253 | 0.266 | 0.275 | 0.024 |
| rs1042714 | 0.426 | 0.439 | 0.036 |

**African/American Population**

| Polymorphism | Male Minor Allele Frequency | Female Minor Allele Frequency | p-value |
| --- | --- | --- | --- |
| rs4673 | 0.45 | 0.24 | 0.037 |
| rs243865 | 0.03 | 0.21 | 0.022 |
| rs213950 | 0.19 | 0.32 | 0.021 |

Hispanic

| Polymorphism | Male Minor Allele Frequency | Female Minor Allele Frequency | p-value |
| --- | --- | --- | --- |
| rs243865 | 0.15 | 0.28 | 0.004 |
| rs6280 | 0.12 | 0.15 | 0.009 |

| Polymorphism | Male Minor Allele Frequency | Female Minor Allele Frequency | p-value |
| --- | --- | --- | --- |
| rs1137101 | 0.43 | 0.27 | 0.045 |
| rs1801133 | 0.13 | 0.29 | 0.036 |
| rs1800872 | 0.43 | 0.63 | 0.046 |
| rs7121 | 0.29 | 0.46 | 0.013 |
| rs4673 | 0.31 | 0.16 | 0.011 |
| rs1799750 | 0.46 | 0.62 | 0.024 |
| rs4691 | 0.26 | 0.41 | 0.025 |

**Asian/Hmong**

Native American

| Polymorphism | Male Minor Allele Frequency | Female Minor Allele Frequency | p-value |
| --- | --- | --- | --- |
| rs1137101 | 0.43 | 0.51 | 0.027 |
| rs1800872 | 0.17 | 0.35 | 0.002 |
| rs1800795 | 0.08 | 0.03 | 0.051 |
| rs4680 | 0.38 | 0.52 | 0.038 |
| rs6313 | 0.48 | 0.34 | 0.011 |
